# HIV Screening among TB Patients and Co-Trimoxazole Preventive Therapy for TB/HIV Patients in Addis Ababa: Facility Based Descriptive Study

**DOI:** 10.1371/journal.pone.0086614

**Published:** 2014-02-03

**Authors:** Amenu Wesen Denegetu, Bethabile Lovely Dolamo

**Affiliations:** 1 STOP International Consultant, WHO South Sudan, Addis Ababa, Ethiopia; 2 Department of Health Studies, University of South Africa, Pretoria, South Africa; Fundacion Huesped, Argentina

## Abstract

**Background:**

Collaborative TB/HIV management is essential to ensure that HIV positive TB patients are identified and treated appropriately, and to prevent tuberculosis (TB) in HIV positive patients. The purpose of this study was to assess HIV case finding among TB patients and Co-trimoxazole Preventive Therapy (CPT) for HIV/TB patients in Addis Ababa.

**Methods:**

A descriptive cross-sectional, facility-based survey was conducted between June and July 2011. Data was collected by interviewing 834 TB patients from ten health facilities in Addis Ababa. Both descriptive and inferential statistics were used to summarize and analyze findings.

**Results:**

The proportion of TB patients who (self reported) were offered for HIV test, tested for HIV and tested HIV positive during their anti-TB treatment follow-up were; 87.4%, 69.4% and 20.2%; respectively. Eighty seven HIV positive patients were identified, who knew their status before diagnosed for the current TB disease, bringing the cumulative prevalence of HIV among TB patients to 24.5%. Hence, the proportion of TB patients who knew their HIV status becomes 79.9%. The study revealed that 43.6% of those newly identified HIV positives during anti-TB treatment follow-up were actually treated with CPT. However, the commutative proportion of HIV positive TB patients who were ever treated with CPT was 54.4%; both those treated before the current TB disease and during anti-TB treatment follow-up.

**Conclusions:**

HIV case finding among TB patients and provision of CPT for TB/HIV co-infected patients needs boosting. Hence, routine offering of HIV test and provision of CPT for PLHIV should be strengthened in-line with the national guidelines.

## Introduction

Globally, an estimated 34.0 million people were living with human immunodeficiency virus (HIV) at the end of 2011. An estimated 0.8% of adults aged 15–49 years worldwide were also living with HIV, although the burden of the epidemic continues to vary considerably between countries and regions [1].

In 2011, people living with HIV (PLHIV) accounted for 1.1 million (13%) of the estimated 8.7 million people who developed TB worldwide and were living with HIV. In addition, of the people with TB who received an HIV test result, 23% tested positive in 2011 alone [2].

TB remains a major global public health problem; it causes ill-health among millions of people each year and ranks as the second leading cause of death from an infectious disease worldwide, after the HIV. In African countries, the proportion of TB cases co-infected with HIV was 39%, which accounted for 79% of TB cases among people living with HIV worldwide [2].

Collaborative TB/HIV management is essential to ensure that HIV positive TB patients are identified and treated appropriately, and to prevent TB in HIV positive people [3]. Activities to decrease the burden of HIV among TB patients include: providing HIV testing and counseling (HTC) to patients with presumptive and diagnosed TB patients, introducing HIV prevention interventions for patients with presumptive and diagnosed TB, providing CPT for TB patients living with HIV, ensure HIV prevention interventions, treatment and care for TB patients living with HIV and provide antiretroviral therapy for TB patients living with HIV [3], [4], [5].

Majority of PLHIV do not know their HIV status and seek health care from general service providers. HTC for people with diagnosed or presumptive TB offers an entry point for a continuum of prevention, care, support and treatment for HIV and for TB [3]. Hence, WHO policy on Provider-Initiated HIV Testing and Counseling (PITC) recommends routine HTC to all patients with presumptive and diagnosed TB as benefits of testing accrue to the patient, their partner, the family and the community at large [4].

The policy statement of the national guideline states, HTC services shall be integrated into existing healthcare services and promoted at all settings. In addition, PITC shall be promoted as part of standard clinical management and care in all health facilities [6], [7]. Besides, the guideline outlines standard operating procedures, which includes, HIV testing is voluntarily with informed consent, confidentiality should be maintained, adequate pre and post test counseling should be offered, test results will be provided in person and service providers need to have proper in-service trainings on HTC [6].

According to the Ethiopian collaborative TB/HIV implementation guideline, HIV testing should be routinely offered to all TB patients at TB clinics. The health worker stationed at TB clinic will be responsible to counsel and refer TB patient for rapid test, if the patient accepts the test. Besides, the decision to be tested for HIV is entirely that of the individual, who must be assured that the process will be confidential [7].

CPT is a broad spectrum antimicrobial agent that prevents a range of secondary bacterial and parasitic infections in eligible adults and children living with HIV [3]. Tuberculosis patients living with HIV should receive CPT and it should be implemented as an integral component of the HIV chronic care package [5].

Evidences from previous studies have shown that CPT reduced mortality, morbidity and hospitalization with no significant increase in adverse events even among smear-positive TB patients with HIV regardless of their CD4 counts [8], [9]. According to the globally 2011 annual TB report, implementation of CPT among TB patients with a documented HIV positive test result reached 79% in 2011 [2].

Information about the status of implementation of collaborative TB/HIV activities to identify HIV positives among TB patients and provision of CPT for TB-HIV co-infected patients in Addis Ababa is limited. Therefore, the purpose of this study was to assess HIV case finding and CPT among TB patients at public health facilities of Addis Ababa, Ethiopia. The findings, as an output, aimed to forward feasible recommendations for policy makers and implementers of TB/HIV care for the city.

### Research Objectives

- To determine the proportion of TB patients who were tested for HIV during anti-TB treatment follow-ups.
- To determine the proportion of HIV positives among TB patients, who were tested during anti-TB treatment follow-ups and before diagnosed for TB.
- To determine the proportion of TB/HIV co-infected patients treated with co-trimoxazole preventive therapy.

## Methods and Materials

### Design

This is a cross sectional facility-based descriptive study which was conducted at two public referral hospitals and eight health centers in Addis Ababa, Ethiopia.

### Settings

The study setting includes *Zewditu* Memorial and *Menelik II* hospitals which are TB/HIV care centers, and ten health centers, namely: *Lideta, Yeka, Kazanchis, Nifas-Silk Lafto No-1, Woreda 7, Kality, Bole* and *Gulele*. The facilities were used to assess the status of implementation of activities to identify HIV positive TB cases and CPT provision among HIV-TB patients attending TB care clinics. In Ethiopia, referral hospitals are expected to have better TB diagnostic and treatment facilities (including sputum exams, x-ray, FNA and inpatient facilities); whereas, health centers are having only sputum microscopy and staffed with medium level health professionals.

### Study Participants

The study participants included TB patients who were enrolled in TB treatment follow-up centers at public health facilities of Addis Ababa. In this study, children of aged 15–17 were interviewed with informed consent from their caretakers/families and the guardians responded for most of past treatment history on their behalf.

### Sampling Method

The sample size was determined using single population proportion formula. The TB/HIV co-infection for Addis Ababa was estimated to be 50%, which gives maximum sample size; marginal error of 5% and 95% confidence interval. The calculated sample size was 384. To eliminate the design effect, the sample size was doubled and 10% of the total sample size was added as contingency. Hence, the total sample size calculated was (384×2) +10% = 845. Randomly selected TB patients attending anti-TB treatment follow-ups at the selected health facilities were interviewed at exit.

In this study, the researcher used probability proportional to size (PPS) of cumulative TB patient load in the facility to determine the subsamples for each of the sites. It was confirmed from TB registration books that, the patient load in health centres on average was less than the load in the hospitals. In addition, the commutative load of patients among the different health centres was similar and that for hospitals was also similar. Therefore, the researcher decided to take an equal number of sub-samples from each of the eight health centres and also equal number from the two referral hospitals. But the sampled population from each of the health centers was less than the samples from the hospitals as reasoned above.

According to the health facilities’ TB registration books, the study health centres and hospitals had on average 550 and 710 cumulative TB patients at the time of the study; respectively. Hence, based on PPS method, the calculated sampled population for each of the health centres was 80 and that for each hospital was 103.

### Data Collection

The data collection was conducted from June to July 2011. The questionnaire used was interviewer administered. It was an adaption from the WHO tool, prepared for monitoring and evaluation of TB/HIV activities [10]. The questionnaire has acceptable levels of reliability and is valid for the Ethiopian situation. The researchers believe that the questionnaire is reliable as data was collected by direct interview of TB patients after their consent and reassurance of confidentiality of information. In addition, the interviews were conducted by sociologists who have no direct relation with the healthcare system and hence avoid professional bias. On top of these, study participants were interviewed at exit of the healthcare services which would reduce recall bias. Ten data collectors (social science graduates) conducted the interviews, and the principal investigator provided supportive supervision during data collection by checking the completeness and consistency of questionnaires.

For this study, the questionnaire was pre-tested on 11 participants by each of the data collectors from two health centers. The pre-test data was collected from facilities not included for this study. During the pre-testing, only editorial errors were found and amended.

Ethical clearance was obtained from the University of South Africa and the Addis Ababa City Administration Health Bureau. A support letter from the Addis Ababa City Administration Health Bureau and permissions from heads of each of the study health facilities was obtained. The interview was undertaken after ascertaining written consents from each study participants. The questionnaire did not include any identifier of the interviewee.

### Data Analysis

The Statistical Package for the Social Sciences (SPSS Inc) version 15.0 was used for data capturing and analysis. Both descriptive and inferential statistics was used for presenting the findings. Logistic regression was used to identify factors associated with offered for HIV among the various independent variables.

The dependent variables used to draw conclusions in this study were: *HIV testing offered and/or tested*, *Prevalence of HIV among TB patients* and Treatment with *CPT*. The independent variables used for associations were: age, marital status, religion, educational status, occupation and being encouraged for HIV test.

## Results

A total of 834 TB patients, who were under anti-TB treatment follow-up in government health facilities in Addis Ababa City Administration, participated in the study. The response rate to the calculated sample size is 98.7%. Out of the total respondents 470 (56.4%) were females.

The mean age (± SD) of the study population was 31.3±11.9 years. Five hundred and eighty (69.6%) of the participants were in the age group of 15–34, 386 (46.3%) were married and 357 (42.8%) were singles, 674 (80.8%) were followers of Christian religion. About half of the participants (50.7%) had completed at least high school education. Two hundred eighty five (34.2%), 231 (27.7%), 147 (17.6%) and 118 (14.1%); were employed by governmental or nongovernmental organizations, unemployed, house wives and self-employed by occupation; respectively (Table 1).

**Table 1:** Socio-demographic and disease characteristics of study participants, Addis Ababa City Administration, July 2011.

Age Group	Frequency	Percent
15–24	259	31.1
25–34	321	38.5
35–44	150	18.0
44+	104	12.5
**Sex**		
Male	364	43.6
Female	470	56.4
**Marital Status**		
Single	357	42.8
Married	386	46.3
Others	91	10.9
**Religion**		
Christian	674	80.8
Muslim	160	19.2
**Ethnicity**		
Amhara	373	44.7
Oromo	220	26.4
Gurage	135	16.2
Others	106	12.7
**Educational Status**		
Illiterate	179	21.5
Primary (1–6)	332	27.8
Secondary (7–12)	344	41.2
Tertiary (12+)	79	9.5
**Occupation**		
Employed (Gov/NGO)	285	34.2
Unemployed	231	27.7
House wife	147	17.6
Self employed/Merchant	118	14.1
Others	53	6.4
**Duration of TB disease**		
Less than 2 months	394	47.2
2–5 months	322	38.6
6–8 months	79	9.5
More than 8 months	39	4.7

Nearly half (47.2%) of the study participants were less than 2 months since diagnosed for active TB disease at the time of interview. All of them were, of course, on their anti-TB treatment in the study health facilities. Slightly higher than half (51.3%) were on their intensive phase treatment period (0–2 months).

### Screening for HIV at TB Clinics

**Figure 1:**
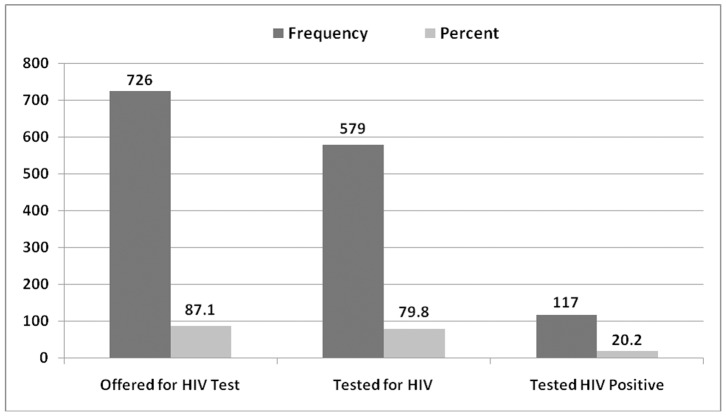
HIV case finding among Tuberculosis patients in Addis Ababa, Ethiopia, August 2011.

The proportion of interviewed TB patients who had been offered for HIV test by TB healthcare provider during any one visit of their anti-TB treatment follow-ups were 87.1% (726). Of those offered for HIV test, 79.8% (579) were tested and 117 (20.2%) tested positive for HIV (Figure 1). In general, 69.4% (579/834) of interviewed TB patients knew their HIV status during anti-TB treatment follow-up. However, 87 TB patients self reported that they had already tested HIV positive before diagnosed for TB disease; hence, the total number of TB patients who knew their HIV status becomes 666 (79.9%); *i.e,* both before the current TB disease and during follow-up.

Multiple logistic regression analysis with stepwise selection was used to examine for any potential effects of the socio-demographic variables including age, marital status, religion, educational status and occupation for statistical significance of being offered for HIV testing during TB cares. However, none of the selected independent variables showed significant differences on both adjusted and unadjusted odds ratio (Table 2).

**Table 2:** Offering of HIV test by TB care provider, Addis Ababa, Ethiopia, July 2011.

Variable	Ever been offered for HIV testduring TB treatment care		Unadjusted OddsRatio(95%CI)	Adjusted OddsRatio(95%CI)
	Yes (726)	No (108)		
**Age Group**				
15–24	217	42	1.00	1.00
25–34	280	41	1.32(0.83–2.11)	1.11(0.66–1.87)
35–44	140	10	2.71(1.32–5.58)	1.98(0.89–4.42)
45+	89	15	1.15(0.61–2.18)	0.95(0.45–2.02)
**Marital Status**				
Single	298	59	1.00	1.00
Married	348	38	1.81(1.72–2.80)	1.60(0.94–2.71)
Others	80	11	1.44(0.72–2.87)	1.46(0.67–3.21)
**Religion**				
Christian	590	84	1.00	1.00
Muslim	136	24	0.81(0.49–1.32)	0.86(0.52–1.44)
**Educational Status**				
Illiterate	154	25	1.00	1.00
Primary (1–6)	196	36	0.88(0.51–1.54)	1.10(0.60–2.03)
Secondary (7–12)	304	40	1.23(0.72–2.11)	1.68(0.90–3.14)
Tertiary (12+)	72	7	1.67(0.69–4.04)	2.23(0. 86–5.76)
**Occupation**				
Employed (Gov/NGO/Priv)	249	36	1.00	1.00
Unemployed	190	41	0.67(0.41–1.09)	0.80(0.48–1.34)
House wife	135	12	1.63(0.82–3.23)	1.66(0.78–3.50)
Self employed/Merchant	105	13	1.17(0.60–2.29)	1.19(0.60–2.36)
Others	47	6	1.13(0.45–2.84)	1.40(0.54–3.63)

### Prevalence of HIV Among Tuberculosis Patients

In this study, 20.2%% (117 out of 579 tested) of study participants have been tested positive for HIV during their anti-TB treatment cares (Figure 1). Nearly, half (47.9%) of these HIV positive TB patients were identified at the same time of being diagnosed for TB or within 2 months. Whereas, 42(35.9%) of all identified HIV positives were detected during 2–5 months of anti-TB treatment follow-up.

In general, a total of 204 HIV positive patients (87 before TB disease and 117 after TB disease) were identified among TB patients who participated in this study. Based on this, the commutative prevalence of HIV positives among all the study TB patients becomes 24.5% (204/834) [Figure 1].

### Co-trimoxazole Preventive Therapy

Only nearly a quarter of, 193 (23.1%), study participants were aware of the benefits of CPT for TB/HIV positive patients. However, majority (91.2%) of those aware of its benefit were also oriented that CPT is provided as part of the package of TB/HIV care at public health facilities in Addis Ababa.

**Figure 2:**
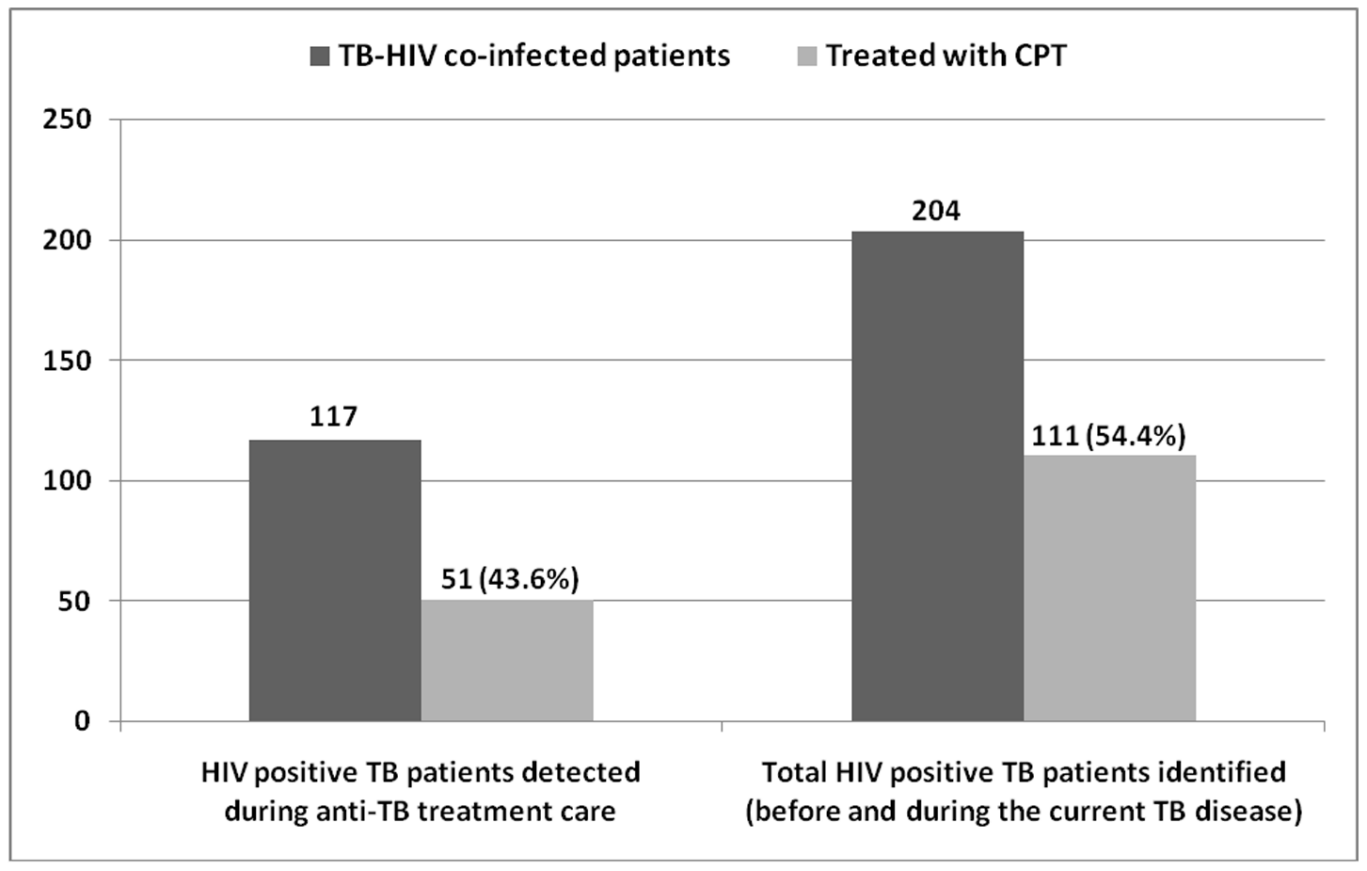
Co-trimoxazole Preventive Therapy among TB-HIV co-infected patients in Addis Ababa, Ethiopia, August 2011.

Hence, the study revealed that 51/117 (43.6%) of the HIV positive TB patients, identified during anti-TB follow-up cares, had ever been treated for CPT. However, 60 TB patients among those who already knew their positive HIV status before their current TB disease (87 HIV positives) had reported being treated with CPT. Hence, out of the total HIV positive TB patients identified (before and during TB treatment), 54.4% (111/204) self reported that they had ever been enrolled for CPT (Figure 2). Majority of the patients refilled their CPT drug from HIV clinics (90/111) and the rest (21/111) from TB clinics.

## Discussion

According to previous report, the number of HIV-infected patients with TB peaked in 2004, with 1.39 million cases, of which, sub-Saharan African countries accounted for nearly 80% of the estimated global burden of HIV infection-associated TB in 2007. South Africa alone accounts for nearly one-third of the global burden [11].

It has been well evidenced that, HIV testing for all TB patients followed by provision of CPT and early initiation of ART for those found to be HIV positive has dramatically reduced mortality among patients with both TB and HIV infection [12].

According to the global TB report 2011, the proportion of notified TB patients who are tested for HIV in the African region has increased from 60% in 2010 to 69% in 2011; 46% of those tested in 2011 were HIV-positive, ranging from 8% in Ethiopia to 77% in Swaziland. In addition 40% of all TB patients notified worldwide in 2011 had a documented HIV test result, up from 33% in 2010 and more than ten times the level of 2004 [2].

In this study, majority (87.1%) of TB patients were offered or encouraged to have HIV test at TB clinics during their anti-TB treatment follow-up. Of these encouraged or offered, 79.8% accepted for the HIV test. However, 87 TB patients self reported that they had already tested HIV positive before diagnosed for TB disease; hence, the total number of TB patients who knew their HIV status until the interview day becomes 666 (79.9%). This finding is slightly higher than two recent reports from USA, (California and New York City) which shows 66% and 70% of TB patients knew their HV status; respectively [13], [14].

However, a retrospective recent study from TB registry in Oromya region of Ethiopia reported a higher rate, where 98% of TB patients had been offered for HIV test and nearly all of them accepted and tested for HIV [15]. On the other hand, this finding is much higher than a recent similar study done in Eastern India, which revealed only one-fourth of patients with TB were tested for HIV during TB treatment follow-up [16].

Therefore, the researchers believe that this finding is not so bad for a country of generalized HIV epidemic status [1]. The reason behind for this high uptake, as observed by the researchers, is utilization of provider initiated HIV Counseling and Testing (PICT) by TB healthcare providers, which entails education and counseling on HIV testing for all TB patients irrespective of their previous HIV test [4]. Hence, TB patients are being offered and encouraged for HIV test in accordance with the national HIV testing protocol by the TB healthcare provider at the TB clinic [6]. According to Addis Ababa City Administration Health Bureau, HTC is being offered with informed consent for all TB patients at public health facilities. In addition, those under the age of 18 years were offered HTC services through the consent of their guardians [17]. Offering for HIV test by TB healthcare providers in Addis Ababa did not show significant differences among the various socio-demographic variables. It can be conclude that, the service provision was equitably to all TB patients.

As HIV is one of the factors attributing in fuelling the TB epidemic [18], screening of all TB patients for HIV increases the uptake of HIV positive patients care and treatment. Testing TB patients for HIV and providing CPT to TB patients living with HIV are mainly the responsibility of national TB control programs [2], [3].

The finding in the current study resulted in a 20.2%% HIV positive rate among those tested during the TB treatment follow-up period. Nearly, half (47.9%) of these HIV positive cases were identified during the intensive phase of therapy (same time or within 2 months of being diagnosed for TB). The second majority (35.9%) were identified during 2–5 months of anti-TB treatment follow-up. This indicates that, majority of TB patients might had been infected with HIV before diagnosed for active TB disease.

This result seems slightly less than a recent finding from two studies in Ethiopia, which reveal 32.2% and 27.2% prevalence rate of HIV among TB patients [15], [19]. In addition, the prevalence in this study is much less than findings from similar studies in other African countries which ranged from 42% to 61%; 42% in Uganda [20], 47.2% in Ghana [21], 61% in Kenya [22] and 56% in Malawi [23]. However, the current result is consistent with a recent global report, which indicates the HIV incidence is decreasing in Ethiopia [2].

In this study, 87 TB patients had self reported that they were already HIV positive before diagnosed for the then TB disease, bringing the total number of HIV positive TB patients to 204; *i.e,* 117 during the then TB disease and 87 before. Therefore, the overall HIV prevalence among TB patients becomes 24.5% (204/834). Despite this, still the prevalence of HIV among TB patients in Addis Ababa was found to be less than previous findings [15], [19], [20], [22], [23].

Co-trimoxazole Preventive Therapy is a simple, well-tolerated and cost-effective intervention which improves quality of life for people living with HIV [5]. The value of CPT in reducing the morbidity and mortality associated with HIV infection has been well documented through earlier clinical studies. In addition, CPT is attributed for up to 46% reduction in mortality among individuals infected with HIV in sub-Saharan Africa [8], [24], [25].

According to the recent annual TB report, globally in 2011 alone, 79% of all TB patients known to have HIV-positive, were provided with CPT [2], which is not very far from the global target 100% that is included in the Global Plan to Stop TB, 2011–2015 [26].

In the current study, 43.6% of the HIV positive TB patients, identified during anti-TB treatment follow-up, have been treated for CPT. However, out of the 87 initially identified HIV positive TB patients, who learnt their status before the then TB disease, 60 had been treated for CPT somewhere. Hence, a total of 111 TB-HIV patients had ever been treated with CPT, which increases slightly the overall CPT coverage to 54.4%. This finding is slightly higher than an earlier study in a referral hospital in North-West Ethiopia, which shows 45.9% of patients eligible for CPT actually received treatment [27].

However, the CPT coverage in the current study in Addis Ababa is less than most of previous reports. For example, a retrospective PLHIV document review in referral hospital in Addis Ababa shows 81% linkage to CPT treatment [28]. Besides, a 10-year review of the scale-up of TB and HIV program collaborative activities in Zambia revealed CPT coverage of 70% in 2010 [29]. Recent global report revealed the African and South-East Asian regions achieved high levels of enrolment on CPT; i.e., 79% and 89% of TB patients known to be living with HIV, respectively [2]. The same report outlined countries that achieved >90% CPT coverage in 2011, which includes Burkina Faso, Burundi, India, Indonesia, Kenya, Lesotho Mozambique, Myanmar, Namibia, Rwanda, Swaziland, Uganda and the United Republic of Tanzania [2]. This shows that, Ethiopia needs a lot to do in scaling up provision of CPT for HIV-infected TB patients.

## Conclusion

It can be concluded that HIV case finding among TB patients and provision of CPT for TB/HIV co-infected patients in Addis Ababa is so bad.

HIV testing is being offered optimally by TB healthcare provider to the majority of TB patients equitably. However, the acceptance of HIV testing is less than expected, *i.e*. 100%. This may need improving the quality of counseling and awareness creation for TB patients, and should be offered as a package of their regular follow-up care. The coverage of CPT in the current study seems to be low compared to most of previous findings in the same area and other countries.

Hence, routine offering of HIV test for all TB patients should be strengthened in-line with the national guidelines. Besides, provision of CPT needs to be scaled up by adopting the national and international guidelines. In addition, continuous support of healthcare providers through on-going refresher trainings, experience sharing forums and supplying with job-aids are recommended.

## Supporting Information

Information S1 **Questionnaire for Tuberculosis Patient Participants, Addis Ababa 2011.**
(PDF)
