# Supplementary material for: HIV Screening among TB Patients and Co-Trimoxazole Preventive Therapy for TB/HIV Patients in Addis Ababa: Facility Based Descriptive Study
Source: PLoS One. 2014 Feb 3;9(2):e86614. doi: 10.1371/journal.pone.0086614 (PMC3911916; doi:10.1371/journal.pone.0086614)
Supplement: Information S1 — Questionnaire for Tuberculosis Patient Participants, Addis Ababa 2011. (PDF) [file pone.0086614.s001.pdf]

Questionnaire for Tuberculosis Patient Participants, Addis Ababa 2011

Name of Health Facility ____

Name of interviewer ____

**Part I: Socio-Demographic Characteristics**		**Quest. No:**	
**Q.No**	**Questions**	**Responses**	**Code**
101	Age (full years)	_________	
102	Sex	1. Male<br>2. Female	
103	Marital status	1. Single<br>2. Married<br>3. Divorced<br>4. Widowed<br>5. Separated	
104	Religion	1. Christian<br>2. Muslim<br>99. Other	
105	Ethnicity	1. Amhara<br>2. Oromo<br>3. Gurage<br>4. Tigre<br>88.Didn't mention<br>99. Other _______________	
106	Educational status	1. No formal education<br>2. Primary (grades 1 - 6)<br>3. Secondary (grade 7 - 12)<br>4. Post secondary (12+)	
107	Occupation	1. Housewife<br>2. Government employee<br>3. Nongovernmental employee	

		4. Private employee<br>5. Self employed/Merchant<br>6. Unemployed<br>99. Other)_______________	
**Part II: Prevalence of HIV among TB patients in the health facilities**			
**Q.No**	**Questions**	**Responses**	**Code**
201	How long have you been diagnosed for TB?	1. Less than 2 months<br>2. 2-5 months<br>3. 6-8 months<br>4. More than 8 months	
202	How long have you been on TB treatment?	1. Less than 2 months<br>2. 2 -5 months<br>3. 6-8 months<br>4. More than 8 months	
203	Have you ever been tested for HIV before you know your TB disease?	1. Yes<br>2. No<br>3. I don't remember/know	
204	If yes to Q203, when have you been tested?	1. Before 6 months<br>2. Before 6-11 months<br>3. Before 1-2 years<br>4. Before >2 years	
205	If yes to Q204, how was your result?	1. Negative<br>2. Positive<br>3. Don't want to disclose	
206	If positive to Q205, have you started of HIV care and treatment?	1. Yes<br>2. No	
207	If No for Q203 or negative to Q205, have you been offered for HIV test now during your TB treatment?	1. Yes<br>2. No	

208	If yes to Q207, have you been tested?	1. Yes<br>2. No	
209	If yes to Q208, who offered the test?	1. TB clinic health worker<br>2. At HIV care/VCT room<br>3. At ANC room<br>4. At outpatient department<br>5. Inpatient department<br>99. Other ________________	
210	If yes to Q207, when have you been tested?	1. During the same time of TB diagnosis<br>2. Within 2 months of TB treatment<br>3. Within 3-5 months during TB treatment<br>4. After 5 months during TB treatment<br>5. I don't remember	
211	If yes to Q208, how was your result?	1. Negative<br>2. Positive<br>3. Don't want to disclose	
212	If positive to Q210, have you started of HIV care and treatment?	1. Yes<br>2. No	
213	If yes to Q211 or Q206, have you already started HAART?	1. Yes<br>2. No	

Part III: Activities to decrease the burden of HIV among TB patients			
**Q.No**	**Questions**	**Responses**	**Code**
301	Is HIV testing offered or encouraged at TB clinic?	1. Yes<br>2. No<br>3. I don't know	
302	Do you know about co-trimoxazole preventive therapy?	1. Yes<br>2. No	
303	Do people living with HIV/AIDS who have also TB have access to CPT as part of the package of care in this health facility?	1. Yes<br>2. No<br>3. I don't know	
304	If the response to Q 303 is no, why not?	1. I don't know<br>2. No drug/expensive<br>3. Care takers don't order<br>99.Other;specify:___________	
305	Have you ever been provided with CPT?	1. Yes<br>2. No	
306	If you were taking or currently under CPT, from where did/do you collect the drug?	1. HIV clinic<br>2. TB clinic<br>3. General pharmacy<br>4. I did/do not take<br>99. Other;<br>specify:_______________	

Thank you!!!
